# Health Equity Analysis of Awareness and Use of GetCheckedOnline, British Columbia’s Digital Intervention for Sexually Transmitted and Blood-Borne Infection Testing in 5 Urban, Suburban, and Rural Communities: Cross-Sectional Survey Study

**DOI:** 10.2196/78561

**Published:** 2026-04-10

**Authors:** Rodrigo Sierra Rosales, Aidan Ablona, Hsiu-Ju Chang, Devon Haag, Heather Pedersen, Catherine Worthington, Daniel Grace, Rod Knight, Devon Greyson, Mark Gilbert

**Affiliations:** 1 School of Population and Public Health, Faculty of Medicine, University of British Columbia, Vancouver, BC, Canada; 2 Clinical Prevention Services, BC Centre for Disease Control, 655 West 12th Avenue, Vancouver, BC, V5Z 4R4, Canada, 1 778-879-2457, 1 604-707-2401; 3 Toronto Public Health, Toronto, ON, Canada; 4 School of Public Health & Public Policy, University of Victoria, Victoria, BC, Canada; 5 Dalla Lana School of Public Health, University of Toronto, Toronto, ON, Canada; 6 École de Santé Publique, Université de Montréal, Montréal, QC, Canada

**Keywords:** testing, health equity, digital health, sexually transmitted infections, human immunodeficiency virus (HIV)

## Abstract

**Background:**

Digital sexually transmitted and blood-borne infection (STBBIs) testing services are used to improve testing access, but might replicate existing social inequities. Previous research has shown that the digital STBBI testing service *GetCheckedOnline* has improved access to testing in British Columbia (BC), Canada. As part of the program’s continuous evaluation, we examined awareness and use of the service in 5 urban, suburban, and rural communities where the program has expanded.

**Objective:**

This study aimed to determine if social location is associated with differences in awareness and use of the service in 5 communities outside Vancouver, BC.

**Methods:**

From July to September 2022, we conducted a cross-sectional survey recruiting (in-person and online) sexually active people aged 16 years or older in 5 urban, suburban, and rural communities where *GetCheckedOnline* had sample collection sites available at the time. We examined differences in awareness and use by age, gender identity, sexual identity, race/ethnicity, education, and income using logistic regression models informed by the Health Equity Measurement Framework.

**Results:**

Of the 1658 participants (n=1058, 63.8% in-person and n=600, 36.2% online), 35.3% (586/1658) were aware of *GetCheckedOnline* and 19.5% (324/1658) had used it. Awareness and use were lower in the first and last age quartiles compared to the second quartile (>38 years: awareness odds ratio [OR] 0.23, 95% CI 0.17‐0.32; use OR 0.19, 95% CI 0.12‐0.28; <25 years: awareness OR 0.39, 95% CI 0.28‐0.53; use OR 0.28, 95% CI 0.18‐0.41). Awareness and use were also lower in the lowest income group compared to the highest (awareness OR 0.39, 95% CI 0.24‐0.65; use OR 0.36, 95% CI 0.20‐0.65). Awareness and use were higher among genderfluid, genderqueer, and nonbinary participants compared to men (awareness OR 2.27, 95% CI 1.63‐3.18; use OR 1.97, 95% CI 1.36‐2.84), transgender compared to cisgender participants (awareness OR 2.17, 95% CI 1.54‐3.06; use OR 2.15, 95% CI 0.46‐3.13), and nonheterosexual compared to heterosexual participants (awareness OR 2.37, 95% CI 1.89‐2.97; use OR 2.53, 95% CI 1.91‐3.38). People of color had higher awareness and use vs White participants (awareness OR 1.74, 95% CI 1.34‐2.26; use OR 2.01, 95% CI 1.48‐2.72). Indigenous participants had higher awareness than White participants (OR 1.65, 95% CI 1.19‐2.20) but no difference in use. Women had similar awareness but lower use compared to men (OR 0.68, 95% CI 0.50‐0.92).

**Conclusions:**

*GetCheckedOnline* is an equitable means of access to STBBI testing for some but not all equity-owed groups in BC. Further adaptations should consider factors such as differences in material circumstances to improve its accessibility for all.

## Introduction

Digital interventions for sexually transmitted and blood-borne infections (STBBIs) testing have been established as a strategy to improve access to testing services [1]. These interventions have been designed using diverse service models to provide access to STBBI testing without needing to visit a health care provider [2]. Compared to services offered at clinics, these interventions are expected to provide lower-cost and more convenient testing options [3]. However, after initial general optimism towards digital interventions as a means to equitably improve health care access, equity concerns have been raised over the past decade regarding the performance of these interventions in the real-world setting [4].

Increasing evidence has suggested that digital interventions can potentially reinforce and even create inequities mediated by complex interactions between intervention characteristics and social and structural determinants of health [5,6]. For example, social and structural determinants affect access to the internet and the affordability of technologies. As such, implementation research on digital STBBI testing interventions has increasingly focused on digital health equity, with reports finding differential use and benefit of these interventions according to systems generating social stratification [7]. Studies reporting differential effects have revealed that age, gender, sexual identity, race and ethnicity, educational attainment, and income can be associated with the uptake of digital STBBI testing services [8-20].

Overall patterns show how uptake is generally higher among women, White people with higher socioeconomic status, urban residents, and heterosexual people [7]. However, most studies are based on program statistics of use and do not collect data within communities. Relying exclusively on program use statistics also does not allow for evaluating intermediate implementation outcomes, such as program awareness, which are essential to understanding uptake patterns (ie, to understand the extent to which not using the service is linked to unawareness) [21].

*GetCheckedOnline* is a free online tool that facilitates STBBI testing by allowing users to generate a laboratory requisition based on their reported sexual activity without visiting a health care provider in British Columbia (BC) [2]. Negative results are available through the website, while those testing positive are contacted by a nurse who connects them to appropriate treatment and care and supports partner notification. It was launched in Vancouver in September 2014, and research suggests *GetCheckedOnline* increases testing frequency and is used by populations facing testing barriers [3,11]. For example, a survey of gay and bisexual men suggested that the service provided a culturally appropriate and safe service to those who had not discussed their sexual identity with providers [22]. *GetCheckedOnline* has since expanded to other, less populated, urban, suburban, and rural communities where implementation outcomes may differ from Vancouver.

In this study, we conducted an analysis of awareness and use of *GetCheckedOnline* in communities outside Vancouver where *GetCheckedOnline* is available. Our objective was to determine if sociodemographic characteristics as markers of social location—including age, gender identity, sexual identity, race/ethnicity, educational attainment, and income—were associated with differences in *GetCheckedOnline* awareness and use. We hypothesized that such differences existed and that higher awareness and use would be found for the most privileged group of each characteristic. We hoped the findings from this survey would identify groups where further promotion or adaptation of the service may be needed.

## Methods

### Theoretical Framework

Our study design was informed by the Health Equity Measurement Framework, which emphasizes the role of social stratification processes at the root of inequities and avoids inferring causal relationships as inherited by individuals [23]. Sociodemographic characteristics were used as markers of social location resulting from social stratification, which supported interpretations of *GetCheckedOnline* adoption in different groups.

### Study Design and Setting

We conducted a survey to evaluate awareness and use of *GetCheckedOnline* in communities where the service expanded with laboratory partners outside Vancouver to other urban, suburban, and rural communities in the province: Victoria, Langford, Kamloops, and Nelson in 2016, Kimberly in 2019, and Maple Ridge in 2020 (see map in Multimedia Appendix 1). The survey instrument included questions about *GetCheckedOnline*, participants’ experiences with STBBI testing, participants’ sexual health experiences, sociodemographic characteristics, and participants’ use of technology. The survey was piloted with 17 people from across BC, including 9 members of our community advisory board. We requested feedback on the questionnaire’s comprehension, readability, and flow and revised it accordingly.

### Recruitment and Data Collection

Recruitment took place from July to September 2022 using in-person and online versions of the survey (to maximize recruitment of people with lower digital literacy or access to technology). Our inclusion criteria were residents of BC, 16 years of age or older, sexually active (defined as oral, vaginal, or anal sex with ≥1 partner in the past year), able to complete the survey in English, and not having completed the survey before. Public health and community organization STBBI leads were identified, and peer researchers were hired for each community. For in-person recruitment, these leads and peer researchers identified common gathering places and venues likely to include individuals facing barriers to accessing STBBI testing. These included 1-time and recurrent community events (eg, Pride festivals and markets) and fixed venues (eg, recreational center). At each venue, peer researchers approached potential participants, described the inclusion criteria, and obtained verbal informed consent with participants self-assessing their eligibility and self-administering the paper survey. Online recruitment occurred through community leaders and local agencies circulating study recruitment information by email, social media (eg, Facebook), and peer researchers putting up posters in venues with QR codes directing to the online survey. Additional recruitment occurred through geotargeted online advertising and promotion in online venues relevant to each community (eg, online forums). The online survey was administered through REDCap (Research Electronic Data Capture; Vanderbilt University) and included measures to prevent automated responses or bots (eg, requiring manual entry of a passphrase included in the survey). The survey was designed to take 10-15 minutes to complete. In-person completion times were not recorded. For the online survey, the median completion time was 12 (IQR 8-20) minutes.

### Study Variables

The implementation outcomes for our study were self-reported awareness and use of *GetCheckedOnline*. Awareness was assessed with the question, “With *GetCheckedOnline*, you can get tested for sexually transmitted infections by printing a lab form or downloading an electronic version on your phone, that you then take to a lab, and then get your results online or by phone. Before today, did you know about *GetCheckedOnline*?” (responses: yes/no). Use was assessed by asking, “Have you been tested through *GetCheckedOnline*?” (responses: yes/no/not sure). The independent equity-relevant sociodemographic variables examined in relation to these outcomes were age, gender identity, sexual identity, race/ethnicity, educational attainment, and self-reported annual income. For annual income, a currency exchange rate of CAD $1=US $0.78 is applicable.

### Data Management

Paper surveys were entered into a database using a double-entry procedure by 2 independent researchers, with discrepancies resolved by a third independent researcher (question discrepancy rate ranged between 0% and 4.9%). In-person and online survey data were combined. Participants reporting age <16 years, 0 sexual partners in the past year, and living in locations outside BC were excluded. Online participants with missing or incorrect passphrases or discrepancies in start and completion times inconsistent with survey time zones were considered likely to be bots or ineligible participants and excluded.

### Statistical Analyses

We modeled awareness and use of *GetCheckedOnline* using logistic regression models to estimate the effect of each of our variables of interest. To build these models, we categorized numeric variables, built mutually exclusive categories for multiple-response survey questions, and selected reference groups. Under our hypothesis, those likely to experience the least barriers to digital STBBI testing services, such as *GetCheckedOnline*, were considered the most privileged group and served as the reference for comparisons. Age categories were based on sample quartiles (<25 years, 25-29 years [reference], 30‐37 years, and >38 years). While older age is typically associated with a privileged social location, we selected the 25‐29 years age group as the reference, as an age range favored by assumptions of digital health literacy (positioning younger people as “digital natives”) and targeting of technology marketing [24]. Gender was studied at 2 levels: the first (dimension 1: gender identity) included the categories of men only (reference), women only, and those identifying as genderfluid, genderqueer, agender, nonbinary, or selecting multiple gender identities (GGANB+); the second level (dimension 2: gender modality [25]) included cisgender (reference) and transgender categories. Sexual identity included those identifying as heterosexual only (reference) and lesbian, gay, bisexual, or other nonexclusively heterosexual identities (LGB+). Race/ethnicity included those identifying as White only (reference), Indigenous, and non-Indigenous racialized people (people of color). Additional details of the question to collect this data and how categories were built are included in Multimedia Appendix 1. Education (graduate degree; reference) and income (≥$80,000; reference) did not require further categorization.

**Figure 1.**
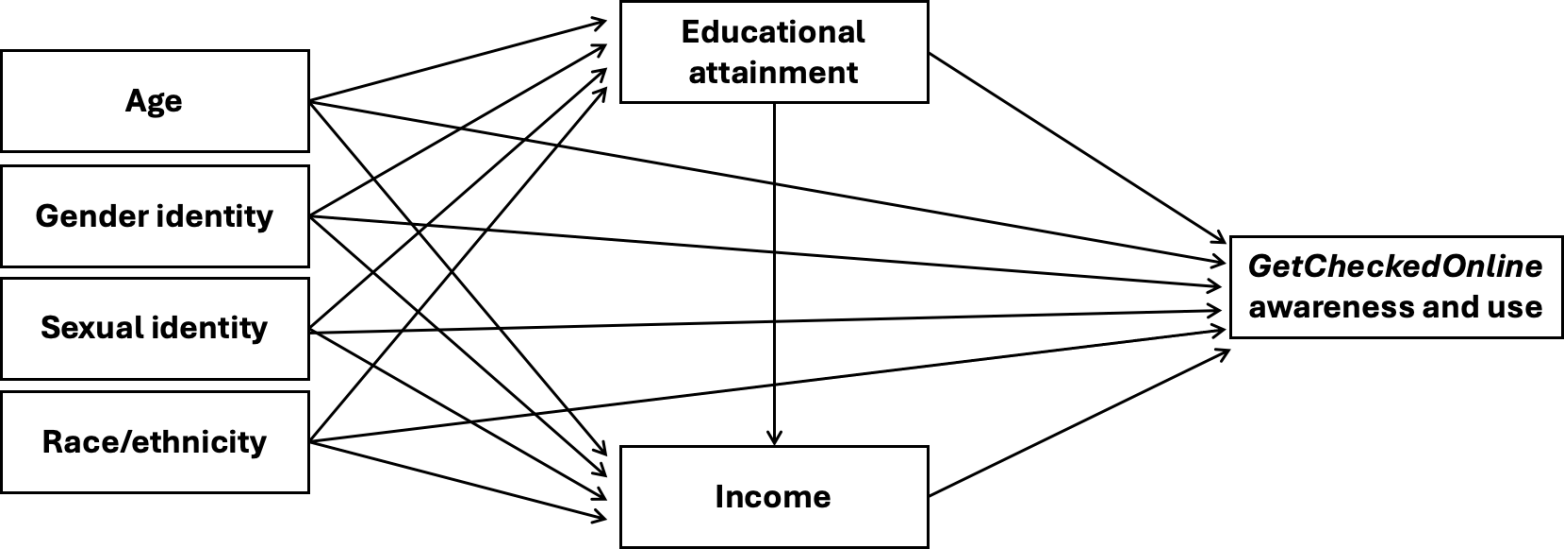
Directed acyclic graph depicting the hypothesized causal pathways between age, gender and sexual identities, race/ethnicity, educational attainment, income, and awareness and use of *GetCheckedOnline*.

Informed by the Health Equity Measurement Framework and the conceptualization of sociodemographic characteristics as proxy variables for multiple social stratification processes [23], we built the directed acyclic graph in Figure 1. We used the DAGitty software (Johannes Textor, Institute for Computing and Information Sciences, Radboud University) to identify the minimal sufficient adjustment set for each of our exposures of interest [26,27]. We built variable-specific models to obtain total direct effects for each exposure [28]. Missing responses were excluded from the analyses. Sensitivity analyses included the assessment of awareness and use distribution comparing those with complete vs missing sociodemographic information using the Fisher exact test to test for differences and identify the potential effects of their exclusion. We report estimated total direct effects through odds ratios (ORs) or adjusted odds ratios (aORs) with 95% CIs. All statistical analyses were conducted using R software (version 4.1.3; R Foundation for Statistical Computing) and its *tidyverse* package [29,30].

### Ethical Considerations

The informed consent procedure was carried out by peer researchers at each venue, who explained the study and obtained verbal consent before handing out surveys to participants. Online and in-person participants completing the survey could enter a draw for 1 of 5 CAD $100 (US $78) VISA gift cards by providing contact information that was not linked to their responses. Participants recruited in person were also offered *GetCheckedOnline* branded promotional materials. Paper survey responses were digitized by 2 core team members and kept with restricted access at the BC Centre for Disease Control. Compiled electronic data from paper and online surveys was stored in secure servers at the BC Centre for Disease Control Central Data Repository. The execution and analysis of the *GetCheckedOnline* community survey were examined and approved by the Behavioral Research Ethics Board of the University of British Columbia (Vancouver, BC, Canada) under the *GetCheckedOnline* Implementation Science Grant (ID H18-00437).

## Results

**Figure 2.**
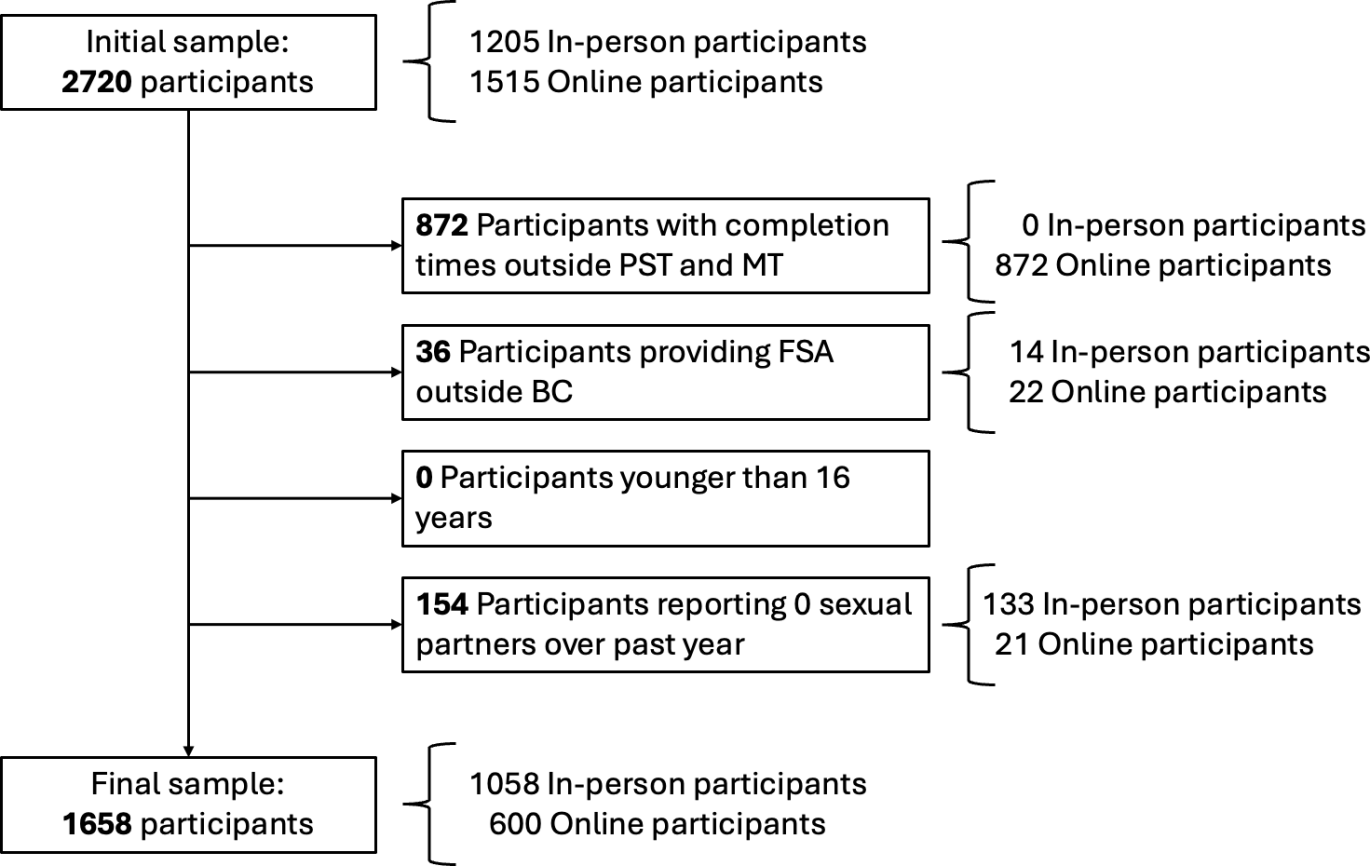
Flowchart of participants included in the analysis of awareness and use of *GetCheckedOnline* in 5 communities outside Vancouver, British Columbia (BC). FSA: Forward Sortation Area of postal code; MT: Mountain Time; PST: Pacific Standard Time.

Overall, 2720 individuals responded to the survey (n=1205, 44.3% in-person and n=1515, 55.7% online). The final sample included 1658 individual participants (n=1058, 63.8% in-person and n=600, 36.2% online; Figure 2). The median age of participants was 30 (IQR 25-38) years (Table 1). Almost half of the participants identified only as a woman (784/1658, 47.3%) and 27.7% (460/1658) only as a man. Three-quarters of the sample identified as cisgender (1246/1658, 75.2%) and 9.1% (151/1658) identified as transgender. More than half of participants identified only as White (945/1658, 57%), followed by Indigenous (209/1658, 12.6%). A total of 39.7% (656/1658) of the sample identified only as heterosexual. Any form of postsecondary education was most common (1062/1658, 64%), and 23.6% (392/1658) reported a personal annual income of less than $22,000 in 2021. More than half of participants (1006/1658, 59.7%) had experienced at least 1 barrier to STBBI testing in the past year (Table 2). Details of participants’ sociodemographic characteristics before mutually exclusive group assignments are included in Multimedia Appendix 1.

Overall, 35.3% (586/1658) of participants were aware of *GetCheckedOnline*, and 19.5% (324/1658) had used the service for STBBI testing. Among those aware (n=586), 55.3% (n=324) had used the service. All independent variables showed statistically significant associations with awareness and use of *GetCheckedOnline* (Table 3). Age and income showed differences in awareness and use, favoring the reference group. For age, when compared to the 25‐29 years group, the remaining age quartiles—particularly the oldest and youngest quartiles—had lower odds of awareness and use (>38 years: awareness OR 0.23, 95% CI 0.17‐0.32; use OR 0.19, 95% CI 0.12‐0.28; <25 years: awareness OR 0.39, 95% CI 0.28‐0.53; use OR 0.28, 95% CI 0.18‐0.41). For income, adjusted for age, gender, sexual identity, race/ethnicity, and education, 2 groups showed statistically significant differences. Compared to the highest income group, those with an income of more than $20,000/year had decreased odds of awareness (aOR 0.39, 95% CI 0.24‐0.65) and use of *GetCheckedOnline* (use aOR 0.36, 95% CI 0.20‐0.65). Compared to the same reference, those with an income between $20,000 and $40,000 per year had similar odds of awareness but lower odds of use (aOR 0.53, 95% CI 0.30‐0.94).

**Table 1.** Sample description and awareness and use of *GetCheckedOnline* by subgroups of participants recruited in 5 communities in British Columbia where the service is available (n=1658).

Variable and subgroup	Sample size, n (%)	Awareness of *GetCheckedOnline*, n/N (%)	Use of *GetCheckedOnline*, n/N (%)
Age			
Q^a^1: <25 years	352 (21.2)	102/352 (29)	40/352 (11.4)
Q2: 25‐29 years	352 (21.2)	182/352 (51.7)	113/352 (32.1)
Q3: 30‐38 years	368 (22.2)	146/368 (39.7)	95/368 (25.8)
Q4: >38 years	403 (24.3)	79/403 (19.6)	33/403 (8.2)
Missing	183 (11)	—^b^	—
Gender—dimension 1 (gender identity)			
Women (only)	784 (47.3)	233/784 (29.7)	114/784 (14.5)
Men (only)	460 (27.7)	159/460 (34.6)	93/460 (20.2)
Genderqueer, genderfluid, agender, nonbinary, and those with multiple gender identities	213 (12.8)	115/213 (54)	70/213 (28.9)
Missing	201 (12.1)	—	—
Gender—dimension 2 (gender modality)			
Cisgender	1246 (75.2)	401/1246 (32.2)	212/1246 (17)
Transgender	151 (9.1)	76/151 (50.3)	45/151 (29.8)
Missing	261 (15.7)	—	—
Sexual Identity			
Heterosexual (only)	656 (39.6)	161/656 (24.5)	77/656 (11.7)
Lesbian, gay, bisexual, and other nonexclusively heterosexual identities	818 (49.3)	354/818 (43.3)	205/818 (25.1)
Missing	184 (11.1)	—	—
Race/ethnicity			
Indigenous	209 (12.6)	88/209 (42.1)	43/209 (20.6)
People of color	311 (18.8)	133/311 (42.8)	86/311 (27.7)
White (only)	945 (57)	288/945 (30.5)	152/945 (16.1)
Missing	193 (11.6)	—	—
Educational attainment			
Less than high school	70 (4.2)	27/70 (38.6)	14/70 (20)
High school	350 (21.1)	100/350 (28.6)	50/350 (14.3)
Postsecondary certificate	517 (31.2)	175/517 (33.8)	98/517 (19)
Bachelor’s degree	395 (23.8)	165/395 (41.8)	95/395 (24.1)
Graduate degree	150 (9)	46/150 (30.7)	24/150 (16)
Missing	176 (10.6)	—	—
Income			
Less than $20,000	392 (23.6)	89/392 (22.7)	41/392 (10.5)
20,000 to 40,000	319 (19.2)	124/319 (38.9)	62/319 (19.4)
40,000 to 60,000	284 (17.1)	123/284 (43.3)	70/284 (24.6)
60,000 to 80,000	179 (10.8)	85/179 (47.5)	59/179 (33)
$80,000 and up	175 (10.6)	57/175 (32.6)	34/175 (19.4)
Missing	309 (18.6)	—	—

a Quartile.

b Not available.

**Table 2.** Usual testing frequency and barriers to provider-based testing for sexually transmitted and blood-borne infections (STBBIs) in a sample of participants from 5 communities in British Columbia where the service is available (n=1658).

Sample descriptor	Value, n (%)
Usual STBBI testing frequency	
Never had an STBBI test	319 (19.2)
Have only tested once	179 (10.8)
No set pattern (including when having symptoms or new sexual partners)	318 (19.2)
Every few years	226 (13.6)
Once a year	227 (13.7)
Twice a year	92 (5.5)
A few times per year (eg, every 3‐4 months)	161 (9.7)
Once a month	18 (1.1)
Missing responses	118 (7.1)
Barriers to provider-based STBBI testing (past year)	
The wait was too long	332 (20)
Needed an appointment	312 (18.8)
Didn’t know where to go	242 (14.6)
The clinic wasn’t open when I could test	212 (12.8)
Concerned about privacy	183 (11)
The clinic was too far away	164 (9.9)
The usual place closed because of COVID-19	149 (9)
Didn’t want to see a doctor or nurse	141 (8.5)
Worried about getting COVID-19 at a testing site	111 (6.7)
Other nonlisted barriers	141 (8.5)
Did not experience barriers to testing in the past year	492 (29.7)

**Table 3.** Associations between sociodemographic characteristics and awareness and use of *GetCheckedOnline* in a sample of participants recruited in 5 communities in British Columbia where the service is available (n=1658).

Sociodemographic characteristic		Awareness, estimate (95% CI)	Use, estimate (95% CI)
Age^a^			
Q^b^1: <25 years		0.39 (0.28‐0.53)^c^	0.28 (0.18‐0.41)^c^
Q2: 25‐29 years		Reference	Reference
Q3: 30‐37 years		0.60 (0.45‐0.81)^c^	0.73 (0.53‐1.01)
Q4: >38 years		0.23 (0.17‐0.32)^c^	0.19 (0.12‐0.28)^c^
Gender^a^—dimension 1 (gender identity)			
Man (only)		Reference	Reference
Woman (only)		0.82 (0.64‐1.04)	0.68 (0.50‐0.92)^c^
GGANB+^d^		2.27 (1.63‐3.18)^c^	1.97 (1.36‐2.84)^c^
Gender^a^—dimension 2 (gender modality)			
Cisgender		Reference	Reference
Transgender		2.17 (1.54‐3.06)^c^	2.15 (1.46‐3.13)^c^
Race/ethnicity^a^			
White (only)		Reference	Reference
Indigenous		1.65 (1.19‐2.20)^c^	1.33 (0.91‐1.93)
People of color		1.74 (1.34‐2.26)^c^	2.01 (1.48‐2.72)^c^
Sexual identity^a^			
Heterosexual		Reference	Reference
LGB+^e^		2.37 (1.89‐2.97)^c^	2.53 (1.91‐3.38)^c^
Educational attainment^f^			
Elementary or some high school		0.79 (0.38‐1.62)	1.39 (0.56‐3.34)
High school		0.84 (0.52‐1.38)	1.09 (0.58‐2.08)
Postsecondary certificate		0.97 (0.62‐1.53)	1.34 (0.76‐2.43)
Bachelor’s degree		1.48 (0.94‐2.35)	1.81 (1.03‐3.31)^c^
Graduate degree		Reference	Reference
Income^g^			
Less than $20,000 per year		0.39 (0.24‐0.65)^c^	0.32 (0.17‐0.58)^c^
$20,000 to <$40,000 per year		0.77 (0.48‐1.25)	0.53 (0.30‐0.94)^c^
$40,000 to <$60,000 per year		0.94 (0.59‐1.49)	0.65 (0.38‐1.13)
$60,000 to <$80,000 per year		1.28 (0.79‐2.09)	1.27 (0.74‐2.20)
$80,000 or more per year		Reference	Reference

a Univariate models: the estimate is an unadjusted odds ratio.

b Quartile.

c Statistically significant results.

d GGANB+: genderqueer, genderfluid, agender, nonbinary people, and those with multiple gender identities.

e LGB+: lesbian, gay, bisexual, and other nonexclusively heterosexual identities.

f Adjusted for age, gender, sexual identity, and race/ethnicity. Estimate is an adjusted odds ratio.

g Adjusted for age, gender, sexual identity, race/ethnicity, and educational attainment. The estimate is an adjusted odds ratio.

In contrast, sexual identity and some comparisons of gender and race/ethnicity showed differences favoring less privileged groups. For sexual identity, higher odds of awareness and use were found in those in the LGB+ group (OR 2.37, 95% CI 1.89‐2.97 for awareness and OR 2.53, 95% CI 1.91‐3.38 for use) as compared to those identifying only as heterosexual. Gender identity (dimension 1) showed higher awareness and use in the GGANB+ group (OR 2.27, 95% CI 1.63‐3.18 for awareness and OR 1.97, 95% CI 1.36‐2.84 for use) compared to men (only). Gender modality (dimension 2) showed higher odds of awareness and use in those identifying as transgender compared to cisgender (OR 2.17, 95% CI 1.54‐3.06 for awareness and OR 2.15, 95% CI 0.46‐3.13 for use). These differences favoring less privileged groups in gender comparisons contrast with what was observed for those identifying only as women, who showed lower odds of use (OR 0.68, 95% CI 0.50‐0.92) but similar levels of awareness compared to men (only).

A similar pattern favoring a less privileged group was observed in the category of people of color as compared to White (only) individuals (OR 1.74, 95% CI 1.34‐2.26 for awareness and OR 2.01, 95% CI 1.48‐2.72 for use). This pattern was not observed among Indigenous individuals who had higher odds of being aware of *GetCheckedOnline* (OR 1.65, 95% CI 1.19‐2.20) compared to White (only) but no difference in use. Lastly, in education, adjusted for age, gender, sexual identity, and race/ethnicity, a difference in use was found between the 2 most privileged groups (bachelor’s degree compared to graduate degree: aOR 1.81, 95% CI 1.03‐3.31) but not lower education groups with no differences in awareness.

In sensitivity analyses, we first compared those who provided complete sociodemographic information (n=1166) to those without sociodemographic information (n=136). Awareness of *GetCheckedOnline* in those who did not give any sociodemographic information (58/136, 42.6%) was not significantly different than in those who provided complete sociodemographic characteristics (420/1166, 36%; Fisher exact test, *P*=.13). Use of *GetCheckedOnline* did differ between these groups (233/1166, 20% vs 39/136, 28.7%; Fisher exact test, *P*=.03). Since more participants skipped the income question than other sociodemographic questions, we compared this group to those who provided complete sociodemographic information in a second sensitivity analysis. Compared to those who provided complete sociodemographic details, those who omitted only the income question (n=109) differed by educational attainment (n=9, 8.3% vs n=45, 3.9% with elementary or some high school education; Fisher exact test, *P*=.02) and race/ethnicity (n=62, 56.9% vs n=796, 68.3% identifying as White; Fisher exact test, *P*=.048). Other sociodemographic characteristics did not significantly differ between these 2 groups. However, the awareness level did not differ between these 2 groups (32/109, 29.4% in those who only omitted income vs 233/1166, 36% in those with complete sociodemographic information; Fisher exact test, *P*=.17). Use of *GetCheckedOnline* did not differ between these groups (14/109, 12.8% vs 233/1166, 20%; Fisher exact test; *P*=.08).

## Discussion

### Principal Results

Awareness and use of *GetCheckedOnline* were high (approximately 1 in 3 and 1 in 5, respectively) in this sample of people in 5 communities where *GetCheckedOnline* is available. We found that differences in sociodemographic characteristics were associated with awareness and use of the service. Some of these differences favored more privileged groups (those in the central quartiles of age and those with higher income). In contrast, other differences favored equity-owed groups (people of color, transgender and gender-diverse participants, and nonheterosexual participants). Two groups—those identifying only as women and Indigenous people—showed similar or higher levels of awareness but not higher use of the service. This study makes an important contribution to the scant but growing literature on the equitable impacts of digital STBBI testing services by being one of the first to measure implementation outcomes across groups at a community level and to use a theoretically informed approach to health equity analyses. This approach has also allowed us to identify the specific equity-owed groups where further promotion of *GetCheckedOnline* may be needed to increase awareness, and where service adaptations may be required to increase use.

### Comparison With Prior Work

Our results align with other research evaluating health equity indicators of digital STBBI testing services, in which differential uptake patterns have been observed with complexity in the directions and magnitude of the effects [7]. Our findings related to age and income are consistent with an overall tendency of interventions favoring privileged groups [7]. However, our findings related to gender, sexual identity, and race/ethnicity follow a different direction, with at least some oppressed groups showing higher uptake. Other studies have reported differences favoring transgender and nonbinary individuals and nonheterosexual men [9,13,20], but all studies finding differences associated with race/ethnicity show higher uptake in White people [9,11,14].

The diffusion of innovations theory proposes concepts that can further support the interpretation of these differences, such as the concept of relative advantage and communication networks [31]. In this case, we examined the relative advantages of *GetCheckedOnline* over provider-based testing in the context of the social stratification processes generating differences in living conditions. Digital access might provide some individuals with an alternative to avoid contact with potential experiences of discrimination in the health care system [32,33], which is a known issue affecting sexual and gender minorities and people of color, even more so in areas away from larger urban centers [34]. Previous research on *GetCheckedOnline* has highlighted the benefits that gay, bisexual, and other men who have sex with men (GBMSM) describe in terms of privacy, control of sample collection, and access to testing by men who live outside of larger urban areas [32]. Transgender and gender-nonconforming individuals have also provided insights into their experiences testing, supporting *GetCheckedOnline*’s adaptation to offer a more inclusive service [35].

In addition, promotional efforts might also explain the higher awareness and use of *GetCheckedOnline* observed in some groups. Early promotion of *GetCheckedOnline* intentionally included representation of and directed marketing to GBMSM [36]. Historical targeting of HIV testing campaigns on GBMSM has also been related to a higher uptake of testing interventions by nonheterosexual men [11]. A higher perception of relative advantage and promotion efforts influences the diffusion through communication networks. This diffusion is more likely to occur among individuals who perceive themselves as similar [31], more so when topics related to sexual health and STBBI still carry a significant social stigma.

However, the service’s relative advantage is impacted by its requirement for digital devices and access to the internet, which has been shown to limit access to digital health services for those with lower incomes [5]. Similarly, *GetCheckedOnline* might be less appealing to those looking for additional services, such as human papillomavirus testing, vaccines, or contraception. In other digital STBBI testing programs, lower uptake in women has been associated with their access to testing in the context of other health-related visits [9]. In other cases, provider-based testing may appear as a more acceptable health care pathway, which might relate to older individuals’ trust in and preference for in-person services. In contrast, *GetCheckedOnline*, as a provincial service, might be perceived as part of the broader health care system, which in turn carries a sense of mistrust from Indigenous communities, coming from the historical and present harms that the colonial systems have perpetrated on them [37]. In addition, they might not see themselves reflected in the service promotion, as *GetCheckedOnline* has not been promoted directly to Indigenous people in the locations included in this study.

### Limitations

Our study had the following limitations. Digital STBBI testing services include diverse service models that might not share essential features of *GetCheckedOnline* (eg, relying on external laboratory partners); therefore, findings might not be generalizable to other digital STBBI testing interventions. Our approach allowed us to estimate the differences associated with the selected sociodemographic characteristics as markers of social location. However, these differences should not be interpreted as the only determinants of the outcomes of our study. Although previous research on *GetCheckedOnline* has shown that variables related to sexual history (eg, behaviors, relationship status, and prior STBBI diagnoses) were not as strongly associated with awareness and use as sociodemographic characteristics [22], these variables may still account for differences in the service implementation outcomes. Similarly, our findings describe the differences associated with markers of social location in these urban, suburban, and rural communities. However, given that geographic location and urbanity function both as materialization and reproduction of social stratification processes [23,38], further analyses and comparisons with larger urban areas may be needed to better understand the role of geographic location on these implementation outcomes.

Furthermore, we relied on self-reported data, which may be subject to volunteer or recall bias, particularly related to implementation outcomes, and some participants did not provide complete sociodemographic information. Sensitivity analyses showed that *GetCheckedOnline* use significantly differed only between those who provided complete sociodemographic information and those who did provide some or no sociodemographic details. However, given the size effect of our found associations and the size of this group, we believe that missing data exclusion would be unlikely to bias the direction of these associations.

### Future Research

Future research can support understanding this complex distribution of service uptake by sociodemographic characteristics. Critical to understanding these uptake patterns is exploring the interactions between different social stratification processes acting simultaneously through an intersectionality lens. Understanding these social processes and focusing on modifiable factors could inform program adjustments. Local context factors at the community level, such as the availability, acceptability, and appropriateness of testing services, play a significant role in service uptake. More research is needed to understand how social stratification processes affect these factors and inform service adaptation as a response. Longitudinal studies can show if the patterns we observed in this study are maintained over time and describe the dynamics of service penetration.

### Conclusions

In conclusion, our study found that patterns of awareness and use of BC’s digital STBBI testing service were associated with sociodemographic characteristics that reflect social stratification processes. Our findings suggest that *GetCheckedOnline* seems to respond equitably to the needs of some equity-owed groups (eg, sexual and gender minorities and people of color). However, further understanding of the lower uptake in other equity-owed groups (eg, lower-income people) is needed. These findings support the need for continuous evaluation of digital STBBI testing programs, and developers should evaluate the factors affecting equity in their local context. Efforts to promote and adapt digital STBBI testing interventions must consider the existing uptake pattern and the social and material circumstances determining accessibility to the service to ensure services reach those who experience a higher burden of these infections.

## Supplementary material

10.2196/78561 Multimedia Appendix 1 *GetCheckedOnline* service availability, GCO Community Survey Questionnaire, and nonmutually exclusive sociodemographic information from survey participants.

## Abbreviations

aOR: adjusted odds ratio
BC: British Columbia
GBMSM: gay, bisexual, and other men who have sex with men
GGANB+: genderfluid, genderqueer, agender, nonbinary, and people with multiple gender identities
LGB+: lesbian, gay, bisexual, or other nonexclusively heterosexual identities
OR: odds ratio
REDCap: Research Electronic Data Capture
STBBI: sexually transmitted and blood-borne infections
