# Supplementary material for: Health Equity Analysis of Awareness and Use of GetCheckedOnline, British Columbia’s Digital Intervention for Sexually Transmitted and Blood-Borne Infection Testing in 5 Urban, Suburban, and Rural Communities: Cross-Sectional Survey Study
Source: J Med Internet Res. 2026 Apr 10;28:e78561. doi: 10.2196/78561 (PMC13068189; doi:10.2196/78561)
Supplement: Multimedia Appendix 1 [file jmir-v28-e78561-s001.docx]

Supplementary Material

Contents

[Appendix A: *GetCheckedOnline* service availability in British Columbia, Canada 2](#_Toc197347135)

[Appendix B: GCO Community Survey Questionnaire 3](#_Toc197347136)

[Appendix C: Additional Non-mutually Exclusive Sociodemographic Information from Survey Participants 13](#_Toc197347137)

## Appendix A: *GetCheckedOnline* service availability in British Columbia, Canada


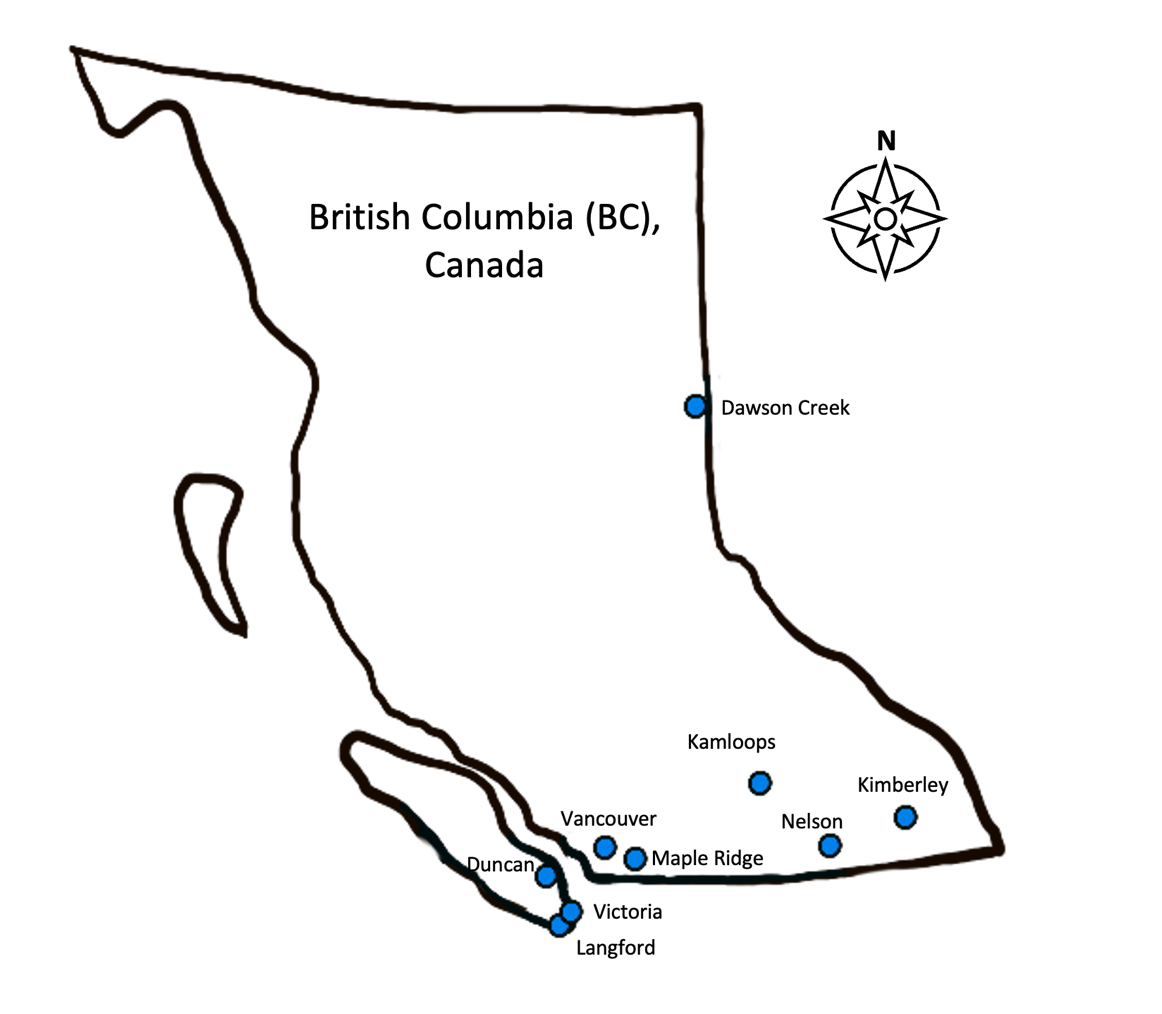


## Appendix B: GCO Community Survey Questionnaire

| **Domain** | **Q#** | **Questions** |
| --- | --- | --- |
| **Part 1: About GetCheckedOnline** | | **Your participation will help us understand experiences with testing services for sexually transmitted infections (STIs) in your community, including GetChekedOnline, a free online STI testing service operated by the BC Centre for Disease Control.** |
| Awareness of GCO  *(Primary Outcome)* | Q1 | **With GetCheckedOnline, you can get tested for STIs by printing a lab form or downloading an electronic version on your phone, that you then take to a lab, and then get your results online or by phone.**  **Before today, did you know about GetCheckedOnline?**  (1) Yes (2) No  ***If No, skip to Q8*** |
| Exposure to promotional channels  *(Community/HA question, user-level)* | Q2 | **How did you hear about GetCheckedOnline?** (check all that apply)  ††(1) News Media (TV, newspaper, etc.) (2) Printed material (posters, brochures, etc.) (3) Ad on a website or phone app (5) Ad on a bus or bus shelter (5) Social media (Facebook, Twitter, etc.) (6) From a physician, nurse, or clinic (7) From someone at a community organization (8) From friends (9) From a relationship/sexual partner (10) Other: _________ (11) I don't remember |
| Use of GCO  *(Primary outcome)* | Q3-4 | **Have you been to the GetCheckedOnline website?**  (1) Yes (2) No (3) Not sure  **Have you been tested through GetCheckedOnline?**  (1) Yes (2) No (3) Not sure |
| Discussions about GCO  *(Secondary outcome)* | Q5-6 | **Did anyone recommend getting tested through GetCheckedOnline to you*?*** *(*check all that apply)  (1)† Friends (2)† †Family (3) Health providers (4) People where I work (5) Relationship/Sexual partners (6) Others not listed (7) No one  **Have you recommended GetCheckedOnline to anyone?**  (1) Yes (2) No (3) Not sure |
| Know someone who has used GCO (*Secondary outcome)* | Q7 | **Do you know anyone who has used GetCheckedOnline?**  (1) Yes (2) No (3) Not sure |
| Intention to use GCO  *(Primary outcome)* | Q8 | **How unlikely or likely is it that you will get tested through GetCheckedOnline in the future?**  (1) Very likely (2) Likely (3) Neither likely nor unlikely (4) Unlikely (5)† Very unlikely |
| Geographical location  *(Socio-demographics, User-level)* | Q9 | **Do you live, work in, or regularly visit any of these communities? (check all that apply)**  (1) City of Victoria (2) Langford (3) Duncan (4) Kamloops (5) Nelson (6) Kimberley (7) Maple Ridge (8) City of Vancouver (9) None of the above |
| Distance to labs  *(Innovation related, user-level)* | Q10 | **With GetCheckedOnline, you have to visit a lab to submit your samples. How much time would you be willing to spend travelling to a lab if you wanted to get tested using GetCheckedOnline?**  (1) Up to 15 min (2) Up to 30 min (3) Up to 45 min (4) Up to one hour (5) More than one hour (6) I would not want to use GetCheckedOnline |
| Perceived benefits & drawbacks of GCO *(Innovation related, user-level)* | Q11-12 | **For you, what are the benefits of using GetCheckedOnline?** (check all that apply)  (1) Don’t need to talk about my sex life (2) Don’t need to get a physical exam (3) Easier to go to a lab than a clinic (4) Getting test results online (5) Testing without waiting for an appointment (6) Testing when the walk-in clinic is full (7) Can use it when I don’t need to see a doctor or nurse (8) Saves time (9) It’s more private (10) Less chance of getting COVID compared to going to a clinic (11) Other: ________ (12) Not sure (13) I see no benefits  **For you, what are the drawbacks of using GetCheckedOnline?** (check all that apply)  (1) Not being able to speak with a doctor or nurse (2) Having to get test results online (3) Finding it hard to get to a lab (4) Feeling uncomfortable going to a lab (5) Not trusting that the service is credible (6) Worrying about the privacy of my online information (7) Needing access to the Internet (8) Not knowning how it works (9) Not being able toget all of the tests I want (10) Other: _____ (11) Not sure (12) I do not see drawbacks |
| **Part 2: Experience with STI testing** | | ***For the following questions, STIs include HIV, syphilis, chlamydia, gonorrhea, and hepatitis C.** |
| Testing history  *(Sexual health care, User leve)* | Q13-14 | **When was your last STI test?**  (1) In the past year  (2) In the last 2 years  (3) In the last 3 to 5 years  (4) More than 5 years ago  (5) Not sure  (6) Never had an STI test - ***Skip to Q16***  **How often do you usually get tested for STIs?**  (1) Have only tested once (2) Every few years (3) Once a year (4) Twice a year (5) A few times per year (e.g. every 3-4 months) (6) Once a month (7) No set pattern (including testing when you have symptoms or new sexual partners |
| Access to clinic-based testing / service availability  *(Sexual health care, User level)* | Q15 | **Where do you usually get tested for STIs? (checked one; only answered by people who have tested)**  (1) A doctor or nurse pratitioner's office (2) A walk-in clinic (3) A STI or sexual health clinic (4) A youth clinic (5) A community health centre (6) A hospital emergency room (7) Through GetCheckedOnline (8) A virtual clinic (9) Some other place; specify _____ (10) No usual place |
| Satisfaction with current testing service  *(Sexual health care, User level)* | Q16 | **Overall, how satisfied or dissatisfied are you with the testing that you receive at your usual place(s) for STI testing?**  (1) Very satisfied (2) Satisfied (3) Neither satisfied nor dissatisfied (4) Dissatisfied (5) Very dissatisfied (6) I do not have a usual place for STI testing |
| Experience of barriers in accessing testing  *(Sexual health care, User level)* | Q17 | **Have any of the following caused you to delay, avoid, or skip testing in the past year?** (check all that apply)  (1) Didn't know where to go (2) Needed an appointment (3) The wait was too long (4) The clinic was too far away (5) The clinic wasn't open when I could test (6) Didn't want to see a doctor or nurse (7) Concerned about privacy (8) Usual place closed because of COVID-19 (9) Worried about getting COVID-19 at a testing site (10) Other: ___ (11) I did not experience barriers to testing in the past year |
| Intention to use home self test kits  *(Sexual health care, User level)* | Q18 | **Suppose you could get tested for STIs by ordering a free and confidential self-collection kit online that would be mailed to any address of your choosing, and then shipped directly to the lab for testing. How likely would it be that you would use this service?**  (1) Very likely (2) Likely (3) Neither likely nor unlikely (4) Unlikely (5)† Very unlikely |
| Sexual health stigma  *(Social, structural level)* | Q19 p1 | **How much do you agree or disagree with the statements below?**  **• It is very embarrassing for me to test for STIs.**  **• I would feel ashamed if someone I knew found out I tested for STIs.**  **• I would feel guilty if I were diagnosed with an STI.**  (1) Strongly agree (2) Agree (3) Neither agree nor disagree (4) Disagree (5) Strongly disagree |
| Empowerment to get tested  *(Sexual health care, User level)* | Q19 p2 | **How much do you agree or disagree with the statements below?**  **• I choose to test for STIs regardless of what other people think.**  **• Testing for STIs is one way I can take charge of my sexual health.**  **• Among my friends, people regularly get tested for STIs.**  (1) Strongly agree (2) Agree (3) Neither agree nor disagree (4) Disagree (5) Strongly disagree |
| Peer norms of testing  *(Social, structural level)* |  |  |
| **Part 3: Your sexual health experiences** | | |
| Usual site of health care access  *(Health care access, User level)* | Q20 | **Where do you usually go for health care when you need care for a minor health problem?**  (1) A doctor or nurse practitioner's office (2) A hospital outpatient clinic (3) A community health centre (4) A walk-in clinic (5) A hospital emergency room (6) A virtual clinic (7) Some other place; specify _________________ (8) No usual place |
| Interaction with healthcare providers (HCPs) during testing  *(Sexual health care, User level)* | Q21 | **In general, how comfortable would it be for you to discuss your sexual history with health care providers?**  (1) Very comfortable (2) Comfortable (3) Neither comfortable nor uncomfortable (4) Uncomfortable (5) Very uncomfortable |
| Prior STI/HIV  *(STBBI-related, user-level)* | Q22-22a | **Have you ever been diagnosed with any of the following STIs? (check all that apply)**  (1) HIV (2) Syphilis (3) Chlamydia (4) Gonorrhea (5) Hepatitis C (6) Another STI diagnosis; specify___ (7) Not sure (8) Prefer not to say (9) I have never been diagnosed with an STI  **If any selected:**  **When was your most recent diagnosis?**  (1) In the past year (2) In the past 2-5 years (3) More than 5 years ago |
| Number of partners  *(STBBI-related, user-level)* | Q23 | **In the last 12 months, how many sex partners have you had?**  (1) 0 (2) 1 (3) 2 (4) 3 (5) 4-5 (6) 6-9 (7) 10 or more (8) Prefer not to say |
| Relationship type  *(STBBI-related, user-level)* | Q24-24a | **Are you currently in a romantic and/or sexual relationship?**  (1) No; ***skip to Q24*** (2) Yes, with one person (3) Yes, with more than 1 person  **Is your current relationship exclusive/monogamous?**  (1) No, we are open (fully or with some rules) (2) Yes, we only have sex with each other (3) Don't know. We haven't discussed it or decided. (4) We don't have sex together (5) Prefer not to say |
| **Part 4 - About You (Demographics)** | | |
| Ethnicity  *(Socio-demographics, User-level)* | Q25-25a | **Which of these groups do you identify with?** (check all that apply)  (1) African (2) Arab and/or West Asian (e.g. Iranian, Afghan) (3) Black (4) Caribbean (5) East Asian (e.g. Chinese, Japanese, Korean) (6) Indigenous (First Nations, Inuit, Métis) (7) Latin American, Hispanic (8) South Asian (e.g. East Indian, Pakistani, Sri Lankan) (9) Southeast Asian (e.g., Filipino, Vietnamese, Thai) (10) White (11) Not listed; please specify ___ (12) Prefer not to say  **If Indigenous:**  **Do you identify as Two-Spirit?**  (1) Yes (2) No (3) Prefer not to say |
| Gender identity  *(Socio-demographics, User-level)* | Q26-27 | **What is your gender identity? (check all that apply)**  (1) Agender (2) Genderfluid (3) Genderqueer (4) Man (5) Non-binary (6) Woman (7) Unsure/questioning (8) I prefer to use another term: _______ (9) Prefer not to say  **What is your gender history/experience?**  (1) My gender is different from my sex assigned at birth (also called transgender) (2) My gender is the same as my sex assigned at birth (also called cisgender) (3) Intersex (4) Prefer not to say |
| Sexual identity  *(Socio-demographics, User-level)* | Q28 | **What best describes your sexual identity?** (check all that apply)  (1) Asexual (2) Bi (bisexual) (3) Gay/lesbian (homeosexual) (4) Heteroflexible (mostly straight) (5) Pansexual (6) Queer (7) Straight (heterosexual) (8) I prefer to use another term: _________ (9) Prefer not to say |
| Education  *(Socio-demographics, User-level)* | Q29 | **What is the highest level of education that you have completed?**  (1) Elementary or some high school (2) High school or equivalent (3) Post-secondary school (e.g., certificate, diploma) (4) Bachelor's degree (5) Graduate degree (Master's, PhD, MD, etc.) (6) Prefer not to say |
| Age  *(Socio-demographics, User-level)* | Q30 | **How old are you?**  (1) _____ years old |
| Geographical location  *(Socio-demographics, User-level)* | Q31 | **What are the first three characters of your postal code?**  (1) ____ (2) Not sure (3) I don't have a permanent address |
| Income  *(Socio-demographics, User-level)* | Q32 | **What was your income (before tax) in 2021?**  (1) Less than $20,000 (2) $20,000-$39,999 (3) $40,000-$59,999 (4) $60,000-$79,999 (5) $80,000 or more (6) Prefer not to say |
| Employment type  *(Socio-demographics, User-level)* | Q33 | **What is your employment status?** (check all that apply)  (1) Employed full-time (30+ hours/week) (2) Employed part-time (less than 30 hours/week) (3) Self-employed (e.g., professional, contractor, business owner) (4) On government assistance (e.g., on disability, E.I.) (5) Student (6) Retired (7) Unemployed (8) Unable to work (9) Prefer not to say |
| Disability | Q34 -34a | **Do you identify as a person with a disability? This can include classification by the government and/or self-diagnosis, and includes all types of disabilities (e.g., physical, mental).**  (1) Yes (2) No (3) Not sure (4) Prefer not to say  **If Yes:**  **Do you have difficulty doing any of the following due to your disability?**  **• Using the Internet**  **• Getting tested for STI in-person**  (1) Yes (2) No (3) Not sure (4) Prefer not to say |
| Housing  *(Socio-demographics, User-level; HA requested)* | Q35-36 | **Have you ever been homeless, that is, having to live in a shelter, on the street or in parks, in a makeshift shelter or in a vehicle or an abandoned building?**  (1) Yes (2) No (3) Not sure (4) Prefer not to say  **Have you ever had to temporarily live with someone else (family, friends, or anyone else) because you had nowhere else to live?**  (1) Yes (2) No (3) Not sure (4) Prefer not to say |
| Drug use  *(equity, user-level)* | Q37-37a | **In the past year, have you used an illegal drug or used a prescription medication for non-medical reasons (for example, because of the experience or feeling it caused)?**  (1) Yes (2) No (3) Not sure (4) Prefer not to say  **If yes, which drugs did you use? (check all that apply)**  (1) Heroin or fentanyl (2) Other opioid products, such as hydromorphone, Dilaudid, Hydromorph Contin, morphine, MS Contin or Demerol (3) Cocaine in any form, including crack, freebase, powder or snow (4) Amphetamines, speed, methamphetamines or crystal meth (5) Ecstasy, also known as MDMA, E, Xtc, Adam or X (6) Hallucinogens such as PCP, LSD, acid, magic mushrooms, Ayahuasca or angel dust (7) Sniffed glue, gasoline or other solvents to get high (8) Tranquilizers or benzos such as valium or xanax (9) Ketamine or GHB (10) Other drug not listed; specify___ |
| **Part 5: Digital Skills and Use of Technology** | | |
| Internet access | Q38 | **How easy or difficult is it for you to go online when you need to?**  (1) Very easy (2) easy (3) neither easy nor difficult (4) difficult (5) Very difficult |
| Digital skills | Q39 | **How often do you use social media websites or apps?**  (1) Several times a day (2) About once a day (3) A few times a week (4) Every few weeks or less often (5) Never |
| Digital literacy | Q40-48 | **For these final questions, we are asking you for your opinions and about your experience using the internet for health information. For each statement, choose the response which best reflects your opinion and experience right now.**  **How important is it for you to be able to access health resources on the Internet?**  R: (1) Very important (2) Important (3) Unsure (4) Not Important (5) Not important at all  **I know what health resources are available on the Internet**  **I know where to find helpful health resources on the Internet**  **I know how to find helpful health resources on the Internet**  **I know how to use the internet to answer questions about my health**  **I know how to use the health information I find on the internet to help me**  **I have the skills I need to evaluate the health resources I find on the internet**  **I can tell high quality health resources from low quality health resources on the internet**  **I feel confident in using information from the internet to make health decisions**  R: (1) Strongly agree (2) Agree (3) Undecided (4) Disagree (5) Strongly Disagree |
| (Online survey only) Recruitment method | Q49 | **How did you get connected to our online survey?**  (1) Got link from someone I know (2) through social media (e.g., Instagram, facebook, twitter) (3) through a community agency (e.g., agency website, newsletter, email) (4) through newsletter (5) printed material about the survey (e.g., QR code, website link) (6) Other way |

## Appendix C: Additional Non-mutually Exclusive Sociodemographic Information from Survey Participants

| **Variable and Description** | | |
| --- | --- | --- |
| **Age** | **Value (years)** | |
| Mean | 33.0 | |
| Standard Deviation | 11.8 | |
| Range | 16 - 85 | |
| Proportion younger than 19 years | 49 / 1,658 (3.0%) | |
| Missing responses | 183 / 1,658 (11.0%) | |
| **Geographic community assigned** | **n** | **%** |
| Kamloops | 234 | 14.1% |
| Kimberley | 177 | 10.7% |
| Maple Ridge | 281 | 16.9% |
| Nelson | 142 | 8.6% |
| Victoria | 494 | 29.8% |
| Other BC region | 108 | 6.5% |
| Did not know FSA or no fixed address | 132 | 8.0% |
| Missing response | 90 | 5.4% |
| **Gender Identity** | **n** | **%** |
| *Dimension 1* *(non-mutually exclusive categories)* |  |  |
| Agender | 31 | 1.9% |
| Genderfluid | 62 | 3.7% |
| Genderqueer | 69 | 4.2% |
| Man | 505 | 30.5% |
| Non-binary | 119 | 7.2% |
| Woman | 840 | 50.7% |
| Unsure | 38 | 2.3% |
| Other | 27 | 1.6% |
| Missing responses | 163 | 9.8% |
| *Dimension 2 (mutually exclusive categories)* |  |  |
| Cisgender | 1246 | 75.2% |
| Transgender | 151 | 9.1% |
| Missing responses | 261 | 15.7% |
| *(Continues on the next page)* |  |  |
| **Race/Ethnicity** *(non-mutually exclusive categories)* | **n** | **%** |
| Indigenous (First Nations, Inuit, Métis) | 209 | 12.6% |
| Black, African, or Caribbean | 119 | 7.2% |
| Middle Eastern, Arab | 49 | 3.0% |
| East Asian | 72 | 4.3% |
| South Asian | 71 | 4.3% |
| Southeast Asian | 46 | 2.8% |
| Latin American | 74 | 4.5% |
| White | 1099 | 66.3% |
| Other | 43 | 2.6% |
| Missing responses | 193 | 11.6% |
| **Sexual Identity** *(non-mutually exclusive categories)* | **n** | **%** |
| Asexual | 49 | 3.0% |
| Bisexual | 324 | 19.5% |
| Gay/Lesbian^^[[1]](#footnote-1)^^ (homosexual) | 138 | 8.3% |
| Heteroflexible (mostly straight) | 195 | 11.8% |
| Pansexual | 159 | 9.6% |
| Queer | 191 | 11.5% |
| Straight (heterosexual) | 705 | 42.5% |
| Other | 21 | 1.3% |
| Missing responses | 184 | 11.1% |
| **Educational Attainment** | **n** | **%** |
| Elementary or some high school | 70 | 4.2% |
| High school or equivalent | 350 | 21.1% |
| Post-secondary school (e.g., certificate, diploma) | 517 | 31.2% |
| Bachelor's degree | 395 | 23.8% |
| Graduate degree (e.g., Master's, PhD, MD) | 150 | 9.0% |
| Missing responses | 176 | 10.6% |
| **Income** | **n** | **%** |
| Less than $20,000 | 392 | 23.6% |
| $20,000-$39,999 | 319 | 19.2% |
| $40,000-$59,999 | 284 | 17.1% |
| $60,000-$79,999 | 179 | 10.8% |
| $80,000 or more | 175 | 10.6% |
| Missing responses | 309 | 18.6% |
| **Usual place to visit for minor health problems** | **n** | **%** |
| Doctor or nurse practitioner's office | 591 | 35.6% |
| Hospital outpatient clinic | 76 | 4.6% |
| Community health centre | 107 | 6.5% |
| Walk-in clinic | 303 | 18.3% |
| Hospital emergency room | 50 | 3.0% |
| Virtual clinic | 104 | 6.3% |
| Other places | 20 | 1.2% |
| No usual place | 67 | 4.0% |
| Missing responses | 340 | 20.5% |
| **Usual STBBI testing frequency** | **n** | **%** |
| Never had an STBBI test | 319 | 19.2% |
| Have only tested once | 179 | 10.8% |
| No set pattern (including when having symptoms or new sexual partners) | 318 | 19.2% |
| Every few years | 226 | 13.6% |
| Once a year | 227 | 13.7% |
| Twice a year | 92 | 5.5% |
| A few times per year (e.g., every 3-4 months) | 161 | 9.7% |
| Once a month | 18 | 1.1% |
| Missing responses | 118 | 7.1% |
| **Barriers to provider-based STBBI testing (past year)** | **n** | **%** |
| The wait was too long | 332 | 20.0% |
| Needed an appointment | 312 | 18.8% |
| Didn't know where to go | 242 | 14.6% |
| The clinic wasn't open when I could test | 212 | 12.8% |
| Concerned about privacy | 183 | 11.0% |
| The clinic was too far away | 164 | 9.9% |
| The usual place closed because of COVID-19 | 149 | 9.0% |
| Didn't want to see a doctor or nurse | 141 | 8.5% |
| Other non-listed barriers | 141 | 8.5% |
| Worried about getting COVID-19 at a testing site | 111 | 6.7% |
| Did not experience barriers to testing in the past year | 492 | 29.7% |
| Missing responses | 175 | 10.6% |

1. Categories were put together in the survey question from which the data was extracted. [↑](#footnote-ref-1)
